# The Culture of Tuberoses

**Published:** 1882-02

**Authors:** William H. Yeomans


### THE CULTURE OF TUBEROSES.

If, as seems to be the case, the farmer's wife is obliged to endure the monotony of farm life, either in the management or execution of household duties it should not only be allowed, but the good housewife should be indulged and assisted in the cultivation of these products of nature, that some one has denominated God's smiles—the beautiful flowers.

There are very many of the most beautiful specimens of the floral world that have been looked upon as beyond the reach of the average farmer's wife and daughter, simply because heretofore, and perhaps almost exclusively, they have been confined to green houses or to the handling of professional florists. Such is the case with the tuberose, a plant of such exquisite bloom and such rich perfume. When the beautiful fragrance of this plant, as it has appeared in the bouquet or floral offering of the professional, has been once tested, there comes a sigh and desire for a still greater and more extensive enjoyment of the luxury. In this there is no difficulty, as it is the purpose of this article to show.

In the first place the plant grows from a bulb, and these can easily be procured in a small quantity for starting, and then the stock can be increased at pleasure. The bulbs increase by enlarged growth from the parent bulb, and three years are required to bring them to bloom, after which the original bulb is of no account, unless to increase the stock, which is hardly desirable, since, with a fair share of care, the developing bulbs will increase the stock with sufficient rapidity.

So far as soil is concerned, any good garden soil that will grow good vegetables will grow tuberoses. It is not necessary that they should be petted and receive all the care and attention that professional florists would claim, because, if kept as they should be, where it is warm and the bulbs slightly moistened for a little time before the soil can be worked, as soon as danger from frost is passed and the soil can be brought into a good mellow condition, operations may be commenced; and if the soil is not of the desired fertility, let any good manure be plowed into the soil and well incorporated with it. Then let the ground be thoroughly worked over and made smooth, and a little furrow marked with the corner of a hoe to the depth of one and a half to two inches, or sufficiently deep to receive the bulbs, which should all be separated, and planted, as near as can be judged, according to the age. It is not desirable to occupy a large amount of space; they may be planted no more than two or three inches apart in the row, and the rows not more than six or eight inches apart, or what is sufficient for the passage of the garden hoe between them. When set, the earth may be hauled carefully over them, and the work of planting is accomplished. Thus the labor of planting is in reality no more than would be necessary to plant potatoes or onions; neither is the after care any more; as they come up, they require occasional hoeing, that the soil may be kept loose, and to prevent the growth of weeds, and in return for this little amount of labor, the laborer will be rewarded with spikes containing from twenty-five to thirty-five blossoms each, with a growth of young bulbs besides. But the care does not quite end here; the plant is extremely susceptible to chilling cold, and the tubers are easily destroyed, therefore it is necessary that, before any severe frosts in the fall, the tubers should be lifted and placed in some warm locality to dry and cure for winter keeping. If some have failed to bloom, that give promise to, such may be lifted and placed in a box of earth and brought into the dwelling, where they will continue to bloom, even into cold weather. When the bulbs are sufficiently dried, they should be stored in a dry, warm place, beyond the possibility of being chilled, and kept until spring. Of course it is desirable, in order to have flowering bulbs each year, to keep a stock of all ages of growth, and then the thing is accomplished.

To show that it is not difficult to make the little bulbs grow, it may be said that last spring, after the family of the writer had planted 3,000 bulbs, they left a number of hundreds of the smaller specimens of the first year's growth, and it occurred to us that a little experiment might be tried touching the difficulty or ease of growing tuberoses; so a small bed was well raked over, and the little bulbs evenly spread over the surface, and lightly covered with loose soil. The result was that they came up thickly, and with a little hand pulling of weeds, did nicely, and this fall were lifted to add to the stock of bulbs of two years' growth. So farmers' wives or daughters need not suppose that the skill of producing tuberoses is confined exclusively to professional florists, for it is a thing which they may possess, if they are so disposed.— *William H. Yeomans, in New England Farmer.*

